# Gastroduodenal Mucosal Injury in Liver Cirrhosis: A Composite Score Analysis of Site-Specific Distribution and Risk Factors

**DOI:** 10.3390/jcm15187094

**Published:** 2026-09-13

**Authors:** Luciana Arjoca, Ana-Maria Filip, Sabrina-Nicoleta Munteanu, Simona Mocan, Sebastian-Ionut Arjoca, Anca Negovan

**Affiliations:** 1 Faculty of Medicine, “George Emil Palade” University of Medicine, Pharmacy, Science, and Technology of Targu Mures, 540139 Targu Mures, Romania; arjoca_luciana@yahoo.com (L.A.); sabrina.munteanu@umfst.ro (S.-N.M.); arjoca_sebastian@yahoo.com (S.-I.A.); ancanegovan@yahoo.com (A.N.); 2 Department of Internal Medicine I, Emergency County Hospital of Targu Mures, 540136 Targu Mures, Romania; 3 Pathology Department, Emergency County Hospital of Targu Mures, 540136 Targu Mures, Romania; slmocan@yahoo.com

**Keywords:** liver cirrhosis, *H. pylori*, gastroduodenal mucosal injury, portal hypertensive gastropathy, Child–Pugh class, gastric corpus, upper gastrointestinal endoscopy

## Abstract

**Background:** Liver cirrhosis is associated with portal hypertension and its gastrointestinal complications, but non-variceal gastroduodenal mucosal injury and its relationship to Helicobacter pylori (*H. pylori*) infection and hepatic disease severity remain incompletely characterized. **Methods:** This retrospective, cross-sectional study included 162 patients undergoing first upper gastrointestinal endoscopy (61 with cirrhosis, 101 controls), assessed using a composite gastroduodenal mucosal injury score (range 0–16) across the antrum, gastric corpus, duodenal bulb, and second duodenal portion. **Results:** Cirrhosis was independently associated with a higher injury burden after adjusting for *H. pylori* status, nonsteroidal anti-inflammatory drug (NSAID), antiplatelet, anticoagulant, and proton pump inhibitor (PPI) use (adjusted count ratio [aCR] = 1.33, 95% CI: 1.09–1.63, *p* = 0.006); and *H. pylori* infection was independently and separately associated with higher injury burden (aCR = 1.25, 95% CI: 1.01–1.54, *p* = 0.037), while none of the four medication classes showed an independent association. This excess injury was concentrated almost exclusively in the gastric corpus (adjusted aCR = 3.04, *p* < 0.0001), with no significant differences at other sites. Injury severity did not correlate with Child–Pugh class or tests of the liver’s biosynthetic capacity (all *p* > 0.05). Only patients with concurrent cirrhosis and *H. pylori* infection showed significantly elevated injury relative to unexposed controls (adjusted aCR = 1.87, 95% CI: 1.38–2.53, *p* < 0.0001), with a significant, exploratory cirrhosis × *H. pylori* interaction (aCR = 1.56, *p* = 0.037). **Conclusions:** Cirrhosis is therefore associated with a corpus-predominant pattern of gastroduodenal injury, compatible with, though not proof of, a portal hypertension-related mechanism, and shows a significant positive multiplicative interaction with concurrent *H. pylori* infection; given the exploratory nature of this analysis, these findings are hypothesis-generating and warrant prospective confirmation before informing endoscopic screening or infection-screening practice.

## 1. Introduction

Liver cirrhosis, the end-stage of progressive hepatic fibrosis with etiologies including alcohol-associated liver disease, chronic viral hepatitis, and steatotic liver disease, remains, alongside hepatocellular carcinoma, a major cause of morbidity and mortality worldwide, accounting for a substantial share of the roughly two million annual liver-related deaths [1,2]. The clinical course of cirrhosis ranges from asymptomatic compensated disease to advanced stages marked by portal hypertension, ascites, hepatic encephalopathy, and gastrointestinal bleeding. Portal hypertension drives the development of gastroesophageal varices, portal hypertensive gastropathy, splenomegaly, and thrombocytopenia, while declining hepatic synthetic and excretory function leads to hypoalbuminemia, coagulopathy, and hyperbilirubinemia as disease progresses [3,4,5].

Upper gastrointestinal endoscopy in cirrhotic patients extends beyond variceal screening to encompass portal hypertensive gastropathy, gastric antral vascular ectasia, peptic ulcer disease, erosive injury, and submucosal hemorrhage, all potential drivers of overt or chronic bleeding. Portal hypertensive gastropathy, typically identified by a mosaic pattern or cherry-red spots, shows variable interobserver classification agreement [6,7,8,9].

Interpreting these lesions is complicated by the comorbidity burden common in cirrhosis, cardiovascular and renal disease, anemia, alcohol use, smoking, and antiplatelet, anticoagulant, or gastrotoxic medication, all of which can affect laboratory findings and mucosal appearance. Dyspeptic symptoms, meanwhile, correlate poorly with either disease severity or endoscopic findings, so many cirrhotic patients undergo their first endoscopy for anemia, thrombocytopenia, or abnormal liver tests rather than classic symptoms.

*H. pylori* infection is a well-established contributor to gastric inflammation, erosive gastritis, and carcinogenesis [10]. Although the antrum is classically the predominant site of *H. pylori*-associated gastritis, corporal, rather than antral, erythema and erosions have been shown to carry stronger discriminative value for infection status, independently predicting *H. pylori* positivity in one endoscopic cohort, whereas antral lesions, despite being more common overall, did not differ significantly between infected and uninfected patients in our previously published cohort [11]. Its specific role in cirrhosis is less clear: studies on its association with portal hypertensive gastropathy report conflicting results, leaving its true contribution to cirrhosis-related mucosal injury unresolved [12,13].

While prior work has focused mainly on variceal bleeding, non-variceal mucosal injury in cirrhosis remains comparatively understudied. It is unclear whether cirrhosis independently increases gastroduodenal injury burden beyond *H. pylori* status and gastrotoxic medication use, whether such injury is diffuse or site-specific, whether it tracks hepatic synthetic dysfunction, and whether cirrhosis and *H. pylori* interact additively or synergistically.

This study compared a composite gastroduodenal mucosal injury score between cirrhotic and non-cirrhotic patients undergoing upper endoscopy, testing whether cirrhosis independently predicts injury burden after adjusting for *H. pylori* status and gastrotoxic medication use (Hypothesis 1), whether injury severity correlates with Child–Pugh class (Hypothesis 2), and whether cirrhosis and *H. pylori* interact synergistically or additively (Hypothesis 3). *H. pylori* was therefore treated as an adjustment covariate for Hypothesis 1 and as a primary exposure for Hypothesis 3, while gastrotoxic medication use served solely as a covariate throughout.

## 2. Materials and Methods

### 2.1. Study Design and Participants

This retrospective, single-center, cross-sectional study included 162 patients who underwent upper gastrointestinal endoscopy at the Internal Medicine Clinic No. 2, County Clinical Emergency Hospital of Târgu Mureș, between 2021 and 2025. The study protocol was approved by the Ethics Committee of the Emergency County Hospital of Targu Mures, Romania (No. Ad. 28994/21 October 2025), and all participants provided written informed consent.

This study is reported in accordance with the STROBE guidelines for observational studies.

Inclusion criteria were: (1) age ≥ 18 years; (2) admission for a medical condition requiring routine blood testing and a first upper gastrointestinal endoscopy during hospitalization; (3) for the cirrhosis group, a diagnosis of liver cirrhosis established by combined clinical, laboratory, and imaging criteria (nodular liver contour and splenomegaly on imaging, together with clinical and laboratory evidence of hepatic dysfunction or portal hypertension); liver biopsy was not used for diagnosis; (4) for the control group, no clinical, laboratory, or imaging evidence of liver cirrhosis; (5) available *H. pylori* status determined by histopathological examination of gastric biopsies.

Patients were excluded if they had: (1) a previous upper gastrointestinal endoscopy during the study period; (2) incomplete medical records; (3) active gastrointestinal bleeding at the time of endoscopy; (4) a history of gastrointestinal malignancy; (5) previous gastric surgery; (6) end-stage cardiac or renal disease; (7) dementia or another condition precluding a reliable clinical history; (8) inflammatory bowel disease; (9) prior *H. pylori* eradication therapy; or (10) cirrhosis status that could not be confidently classified into either group.

### 2.2. Clinical Data Collection

Demographic and clinical data were collected through medical record review. Variables included age, sex, current medications, smoking status, and alcohol consumption history. Chronic medication use was defined as ongoing treatment for at least one month before endoscopy.

NSAID, antiplatelet, anticoagulant, and PPI use were recorded and analyzed as four separate binary covariates, given their distinct mechanisms of gastroduodenal mucosal injury; a combined “gastrotoxic medication” variable (NSAIDs, antiplatelet agents, and anticoagulants merged into a single binary exposure) was also constructed and used only in a robustness-check analysis and in the site-specific and four-group interaction models (Section 3.3 and Section 3.5), where the smaller stratified cell sizes favored a more parsimonious covariate set.

Smoking status was categorized as never/former/current, and alcohol consumption was classified as regular use if ≥1 standard unit on ≥3 occasions per week. Comorbidities were recorded from documented diagnoses and included diabetes, chronic kidney disease, cardiovascular disease, respiratory disease, neoplasia.

### 2.3. Laboratory Assessments

Venous blood samples were collected for routine laboratory analyses. Hemoglobin, international normalized ratio (INR), total serum bilirubin, and serum albumin were measured using standard automated analyzers and used in the primary analysis. *H. pylori* status was determined by histopathological examination of gastric biopsies obtained during endoscopy, using the following histological staining: Hematoxylin and eosin for general morphological analysis, periodic acid-Schiff-Alcian blue for the identification of intestinal metaplasia, and Giemsa stain for the detection of *H. pylori* microorganisms. In cases with persistent histological inflammation but no visible organisms on standard staining, immunohistochemistry was performed, as this method can identify *H. pylori* antigen even when bacterial density is reduced by proton pump inhibitor or antibiotic use, thereby reducing the risk of false-negative diagnosis in such cases.

### 2.4. Endoscopic and Histopathological Assessment

Upper gastrointestinal endoscopies were performed by one specialized endoscopist with experience using high-definition endoscopes (Olympus EVIS EXERA XI systems, Olympus Corp., Tokyo, Japan). Upper gastrointestinal endoscopic findings were systematically recorded for each patient using a pre-specified composite gastroduodenal mucosal injury score, designed to capture the overall burden of mucosal damage independent of any single lesion type or anatomical site. Four sites were assessed at each endoscopy: the antrum, the gastric corpus, the duodenal bulb, and the second duodenal portion (D2). At each site, the presence or absence of four lesion types was recorded independently: erythema/edema, erosions, ulcers, and submucosal hemorrhage. Each lesion type present at a given site contributed one point, yielding a site-specific sub-score ranging from 0 to 4. The four site-specific sub-scores were then summed to generate the total composite score, ranging from 0 (no lesions at any site) to 16 (all four lesion types present at all four sites). This composite score, and its four site-specific sub-scores, served as the primary outcome measure for the statistical analyses described in Section 2.5.

Endoscopic findings were extracted retrospectively from the written endoscopy reports on file for each patient; the scoring criteria for the composite injury score (specifically, which lesion types and anatomical sites would be scored, and how) were pre-specified before data extraction began, in order to standardize coding and minimize the risk of extraction bias. The score itself was not recorded prospectively during the index endoscopy.

Blinding to cirrhosis status varied across patients. In those with an established clinical diagnosis of cirrhosis prior to endoscopy, the endoscopist was aware of this status. However, some patients underwent endoscopy as part of the diagnostic workup for anemia or dyspepsia, and suspicion of cirrhosis arose only from endoscopic findings suggestive of portal hypertension identified during the procedure itself; for these patients, cirrhosis status was therefore not known beforehand. *H. pylori* status, determined histologically after the procedure, was not known at the time of endoscopy for any patient. Five gastric biopsies were obtained and examined from each patient in accordance with the protocol outlined in the updated Sydney System (two antral, two corporal, and one angular sample). Histopathological variables recorded included *H. pylori* status, gastritis and gastropathy pattern, inflammatory activity, glandular atrophy, and intestinal metaplasia.

The composite injury score used in this study was not previously validated as a diagnostic instrument; rather, it was developed as a pragmatic research tool to capture overall mucosal injury burden across multiple lesion types and anatomical sites within a single outcome measure, given the absence of an established, validated composite index for this purpose. Equal weighting of erythema/edema, erosions, ulcers, and submucosal hemorrhage was chosen as a simplifying assumption for hypothesis-generating analysis, rather than reflecting an assumption that these lesion types carry equivalent clinical severity.

These four lesion types were studied because of their recognized potential for non-variceal gastrointestinal hemorrhage in cirrhotic patients, complementing the well-established focus on variceal bleeding risk in this population.

Internal consistency of the composite score, assessed post hoc via the Kuder–Richardson formula 20, was modest (KR-20 = 0.31), consistent with its intended function as a burden count across biologically heterogeneous lesion types and sites rather than a unidimensional severity construct.

Formal inter-rater reliability was not assessed; this represents an acknowledged limitation (Section 4.4).

Recent work has highlighted the potential of AI-assisted and other objective approaches to standardize endoscopic lesion assessment and reduce operator-dependent variability [14], and future validation of composite injury scores of this type could benefit from such tools.

### 2.5. Statistical Analysis

Analyses were performed in R (version 4.3.0; R Foundation for Statistical Computing, Vienna, Austria) within RStudio version 2025.05.1+513 (Posit Software, PBC, Boston, MA, USA), using tidyverse (v2.0.0), gtsummary (v1.7.2), MASS (v7.3-60, for negative binomial regression), and ggplot2 (v3.4.4). Continuous variables were assessed for normality (Shapiro–Wilk test) and presented as mean (SD) or median (IQR) accordingly, compared by *t*-test or Mann–Whitney U as appropriate; categorical variables were presented as *n* (%) and compared by chi-square test, or Fisher’s exact test where expected cell counts were small.

The composite injury score, an over-dispersed count variable, was modeled using negative binomial regression; unadjusted comparisons used the Wilcoxon rank-sum test. For Hypothesis 1, a multivariate negative binomial model tested cirrhosis status as a predictor of injury score, with *H. pylori* status, NSAID use, antiplatelet use, anticoagulant use, and PPI use retained a priori as separate covariates (reported as adjusted count ratios—aCR—with 95% CIs), given their established relevance to mucosal injury, regardless of baseline balance.

Two sensitivity analyses were pre-specified: (i) a fully adjusted model additionally including age, sex, smoking status, alcohol use, and comorbidities (diabetes, chronic kidney disease, cardiovascular disease); and (ii) a subgroup analysis restricted to cirrhotic patients without alcohol-related etiology, to assess the influence of the predominantly alcohol-related etiology in this cohort.

Overdispersion was assessed via the Pearson chi-square/degrees-of-freedom ratio and a likelihood-ratio test comparing the negative binomial to a Poisson model. Individual lesion components were analyzed by site using Firth-penalized logistic regression with Benjamini–Hochberg correction, and lesion-type sub-scores were analyzed across sites using the same approach. Internal consistency of the composite score was assessed with the Kuder–Richardson formula 20 (KR-20).

Site-specific sub-scores (antrum, corpus, bulb, D2) were analyzed identically, with Benjamini–Hochberg correction across sites. For Hypothesis 2, the composite score was compared across Child–Pugh classes using the Kruskal–Wallis test (Jonckheere–Terpstra for ordered trend), and correlated with albumin, INR, and bilirubin via Spearman’s rho; as an internal consistency check, these three markers plus hemoglobin were each compared across Child–Pugh classes (Kruskal–Wallis).

For Hypothesis 3, patients were stratified into four groups (control/HP−, control/HP+, cirrhosis/HP−, cirrhosis/HP+) and compared descriptively (Kruskal–Wallis with Dunn’s post hoc, Benjamini–Hochberg corrected). The primary, confounder-adjusted analysis used a negative binomial model on the same four-group structure (reference: control/HP−), reported as adjusted aCRs with 95% CIs, with a cirrhosis × *H. pylori* interaction term added to test for departure from a multiplicative combination of effects; this analysis was treated as exploratory given the small co-exposed subgroup.

All tests were two-sided; *p* < 0.05 was considered significant, except where noted above.

## 3. Results

### 3.1. Study Population and Baseline Characteristics

**Figure 1:**
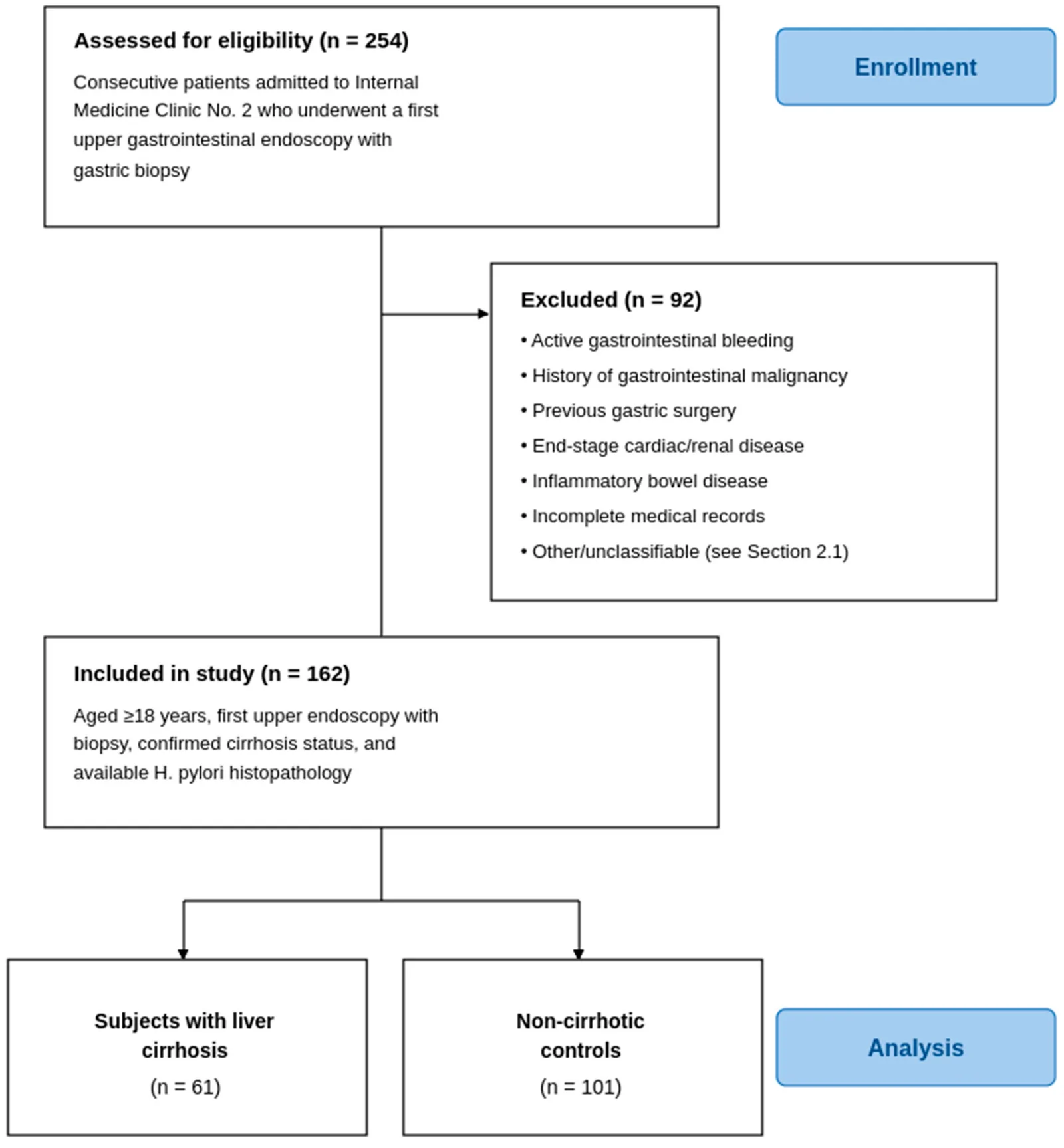
Study population selection.

Of 254 patients initially screened, 92 were excluded for the reasons mentioned above, yielding the final analytic population of 162 patients: 61 with liver cirrhosis and 101 controls (Figure 1). Within the cirrhosis group, disease severity was classified according to the Child–Pugh score (serum bilirubin, serum albumin, INR, and the clinical grading of ascites and encephalopathy at admission): 30 patients were class A, 24 class B, and 7 class C. Cirrhosis etiology was recorded per patient, allowing more than one etiology, and included 52 alcohol-related, 9 viral hepatitis B/C, 0 metabolic dysfunction-associated steatotic liver disease, and 3 other/mixed.

This cross-sectional, single-center study included 162 patients who underwent upper gastrointestinal endoscopy with paired histological assessment: 61 patients with liver cirrhosis and 101 non-cirrhotic controls. The two groups were compared for demographic, clinical, and laboratory characteristics before proceeding to the hypothesis-driven analyses (Table 1).

Cirrhotic patients were significantly older than controls (65.0 vs. 59.7 years, *p* = 0.037) and were more frequently male (68.9% vs. 38.6%, *p* < 0.001). Hemoglobin was notably lower in cirrhotic patients (10.4 vs. 12.2 g/dL, *p* < 0.001).

*H. pylori* positivity was numerically lower in the cirrhosis group than in controls (19.7% vs. 27.7%), although this difference did not reach statistical significance (*p* = 0.267). Similarly, the use of NSAIDs, antiplatelet agents, or anticoagulants was numerically lower in cirrhotic patients but did not differ significantly between groups (27.9% vs. 43.6%, *p* = 0.065).

**Figure 2:**
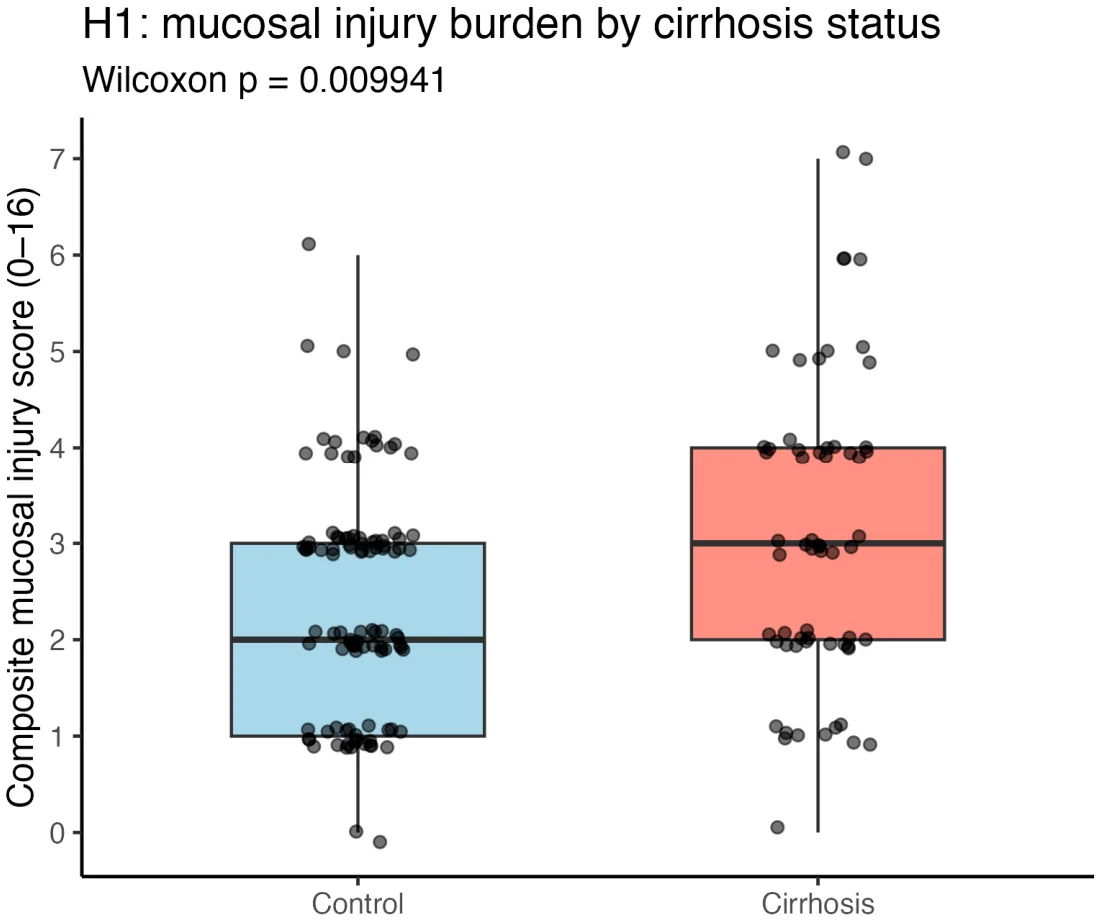
Composite mucosal injury score by cirrhosis status. Boxplots show the composite mucosal injury score (range 0–16) in cirrhotic patients (*n* = 61) and non-cirrhotic controls (*n* = 101). Points represent individual patients; the horizontal line within each box denotes the median, box limits denote the interquartile range (25th–75th percentile), and vertical whiskers extend to the most extreme values within 1.5× the interquartile range from the box edges. The between-group difference was assessed with the Wilcoxon rank-sum test (*p* = 0.0099).

The primary outcome of this study, the composite gastroduodenal mucosal injury score, was significantly higher in cirrhotic patients than in controls at the unadjusted, univariate level (mean 3.11 vs. 2.44; median 3 vs. 2), a difference confirmed by the Wilcoxon rank-sum test (*p* = 0.0099) (Table 1, Figure 2). As shown in Figure 2, although both groups displayed a wide range of individual scores, the cirrhosis group was shifted toward higher values, with a higher median and a visibly greater proportion of patients clustered at scores of 4 and above.

### 3.2. Cirrhosis Is Independently Associated with Increased Gastroduodenal Mucosal Injury (Hypothesis 1)

A multivariate negative binomial regression model was constructed, with the composite injury score as the outcome and cirrhosis status, *H. pylori* status, NSAID use, antiplatelet use, anticoagulant use, and PPI use entered simultaneously as predictors.

Overdispersion was assessed via the Pearson chi-square/degrees-of-freedom ratio (0.65) and a likelihood-ratio test comparing the negative binomial to a Poisson model (*p* = 0.50, θ ≈ 70,317), indicating no meaningful departure from a Poisson process and supporting the appropriateness of this modeling approach.

In this model, the presence of cirrhosis remained a statistically significant and independent predictor of increased mucosal injury burden, with an adjusted count ratio (aCR) of 1.33 (95% CI: 1.09–1.63, *p* = 0.006). *H. pylori* infection was also independently associated with higher injury scores (adjusted aCR = 1.25, 95% CI: 1.01–1.54, *p* = 0.037). None of the four medication classes—NSAIDs (aCR = 1.09, *p* = 0.62), antiplatelet agents (aCR = 0.96, *p* = 0.74), anticoagulants (aCR = 0.97, *p* = 0.84), or PPIs (aCR = 0.96, *p* = 0.70)—showed a significant independent association with injury burden (Table 2).

Results were materially unchanged when NSAID, antiplatelet, and anticoagulant use were combined into a single gastrotoxic-medication variable, as in earlier drafts (aCR = 1.30, 95% CI: 1.07–1.57, *p* = 0.008 for cirrhosis; aCR = 1.24, *p* = 0.041 for *H. pylori*), confirming the finding is not an artifact of how medication exposure was coded.

A sensitivity analysis additionally adjusting for age, sex, smoking history, alcohol use, and comorbidities (diabetes, chronic kidney disease, cardiovascular disease) yielded a similar or slightly larger point estimate for cirrhosis (adjusted aCR = 1.35), though the wider confidence interval associated with this more heavily parameterized model reduced statistical significance to a borderline level (*p* = 0.059).

As a complementary cross-sectional effect measure, a binary logistic model (composite score ≥4 vs. <4) yielded adjusted odds ratios of 4.41 (95% CI 1.98–9.83, *p* < 0.001) for cirrhosis and 3.85 (95% CI 1.66–8.95, *p* = 0.002) for *H. pylori*, consistent in direction and significance with the count-based model above.

Smoking was reported in 24.6% of cirrhotic patients versus 12.9% of controls (*p* = 0.08), and individual drug-class frequencies are given above (NSAID, aspirin, clopidogrel, vitamin K antagonist, and NOAC use).

### 3.3. Mucosal Injury in Cirrhosis Is Concentrated in the Gastric Corpus

Site-specific and four-group interaction models retain the combined gastrotoxic-medication covariate rather than the four separate drug classes, to preserve statistical stability given the smaller cell sizes in these stratified analyses (e.g., *n* = 12 in the cirrhosis/*H. pylori*-positive subgroup).

The injury sub-score at each of the four sites (antrum, corpus, duodenal bulb, and second duodenal portion) was analyzed separately, using negative binomial regression adjusted for *H. pylori* status and NSAID/antiplatelet/anticoagulant use, with the resulting four *p*-values corrected for multiple comparisons using the Benjamini–Hochberg procedure.

**Figure 3:**
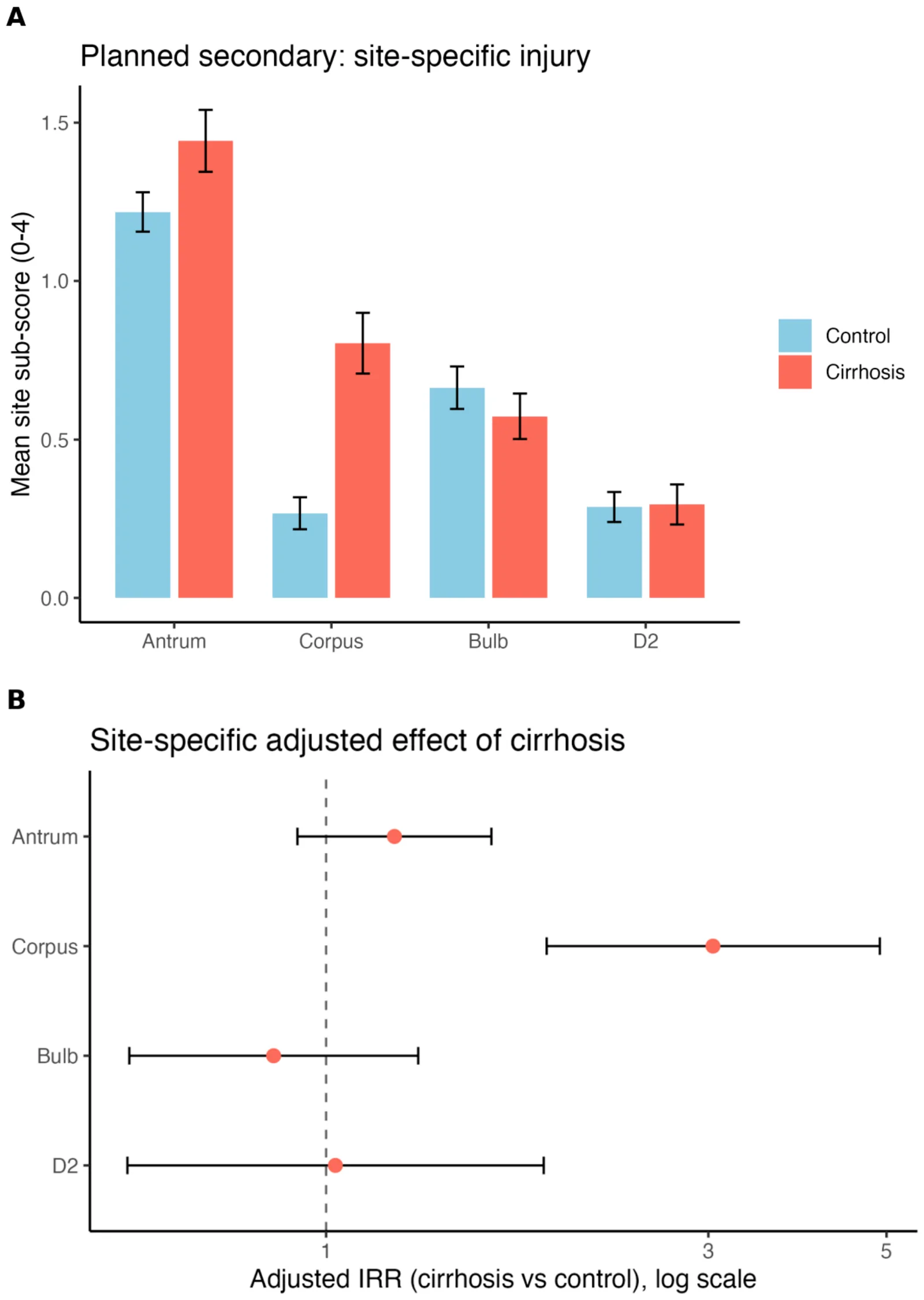
Site-specific gastroduodenal mucosal injury by cirrhosis status. (**A**) Mean site-specific injury sub-scores (range 0–4) for the antrum, corpus, duodenal bulb, and second duodenal portion (D2), by group; error bars represent the standard error of the mean. (**B**) Forest plot of adjusted incidence rate ratios (IRR) for cirrhosis versus control at each anatomical site, derived from negative binomial regression models adjusted for *H. pylori* status and NSAID/antiplatelet/anticoagulant use. Horizontal lines represent 95% confidence intervals; the dashed vertical line indicates an of 1 (no effect); the x-axis is on a logarithmic scale. *p*-values were corrected for multiple comparisons (Benjamini–Hochberg, four comparisons).

At the gastric corpus, the mean injury sub-score nearly tripled in cirrhotic patients relative to controls (0.80 vs. 0.27), corresponding to an adjusted aCR of 3.04, a difference that remained highly significant even after correction for multiple testing (*p* < 0.0001). In contrast, none of the remaining three sites showed a significant between-group difference: the antrum (adjusted = 1.22, corrected *p* = 0.34), the duodenal bulb (adjusted = 0.86, corrected *p* = 0.64), and the second duodenal portion (adjusted = 1.03, corrected *p* = 0.93) all showed injury sub-scores that were statistically indistinguishable between cirrhotic patients and controls (Table 3, Figure 3).

To determine which lesion types underlie the corpus-predominant finding, we analyzed the four individual lesion components (erythema/edema, erosions, ulcers, submucosal hemorrhage) at each site using Firth-penalized logistic regression with Benjamini–Hochberg correction across the 16 components tested (Table 4). This corpus signal was driven predominantly by corpus erythema/edema (adjusted OR = 5.96, 95% CI: 2.81–13.32, corrected *p* < 0.0001), a mild and nonspecific finding; corpus submucosal hemorrhage showed a numerically higher odds ratio (OR = 2.91) that did not survive correction for multiple comparisons (corrected *p* = 0.16), and corpus erosions and ulcers showed no significant between-group difference (corpus ulcers had zero events in either group). Across all four sites combined, submucosal hemorrhage (=2.48, corrected *p* = 0.06) and erosions (=1.58, corrected *p* = 0.12) trended higher in cirrhotic patients, approaching but not reaching significance after correction, while erythema and ulcers showed no such trend. We interpret this pattern as indicating that the corpus-predominant excess is driven mainly by mild mucosal change rather than by ulceration or overt hemorrhage, which tempers, without negating, its clinical significance.

### 3.4. Mucosal Injury Does Not Correlate with Child–Pugh Class or Hepatic Synthetic Function (Hypothesis 2)

This was tested in two complementary ways: first, by comparing the composite injury score across the three Child–Pugh classes (A, B, and C); second, by directly correlating the injury score with three continuous markers of hepatic synthetic and coagulation function (albumin, INR, and total bilirubin).

The composite injury score did not differ significantly across Child–Pugh classes A, B, and C (mean 3.23, 2.92, and 3.29, respectively). This was confirmed both by the Kruskal–Wallis test, which compares the three groups without assuming any particular ordering (*p* = 0.885), and by the Jonckheere–Terpstra test, which is specifically designed to detect an ordered trend across groups (A < B < C or the reverse) and would have been more sensitive had a true gradient been present (*p* = 0.448). Neither test suggested any relationship between Child–Pugh class and injury burden. When the four anatomical sites were examined individually, no site-specific sub-score showed a significant trend across Child–Pugh classes either, indicating that this null result was not masked by opposing trends at different sites that might have canceled out in the composite score.

**Figure 4:**
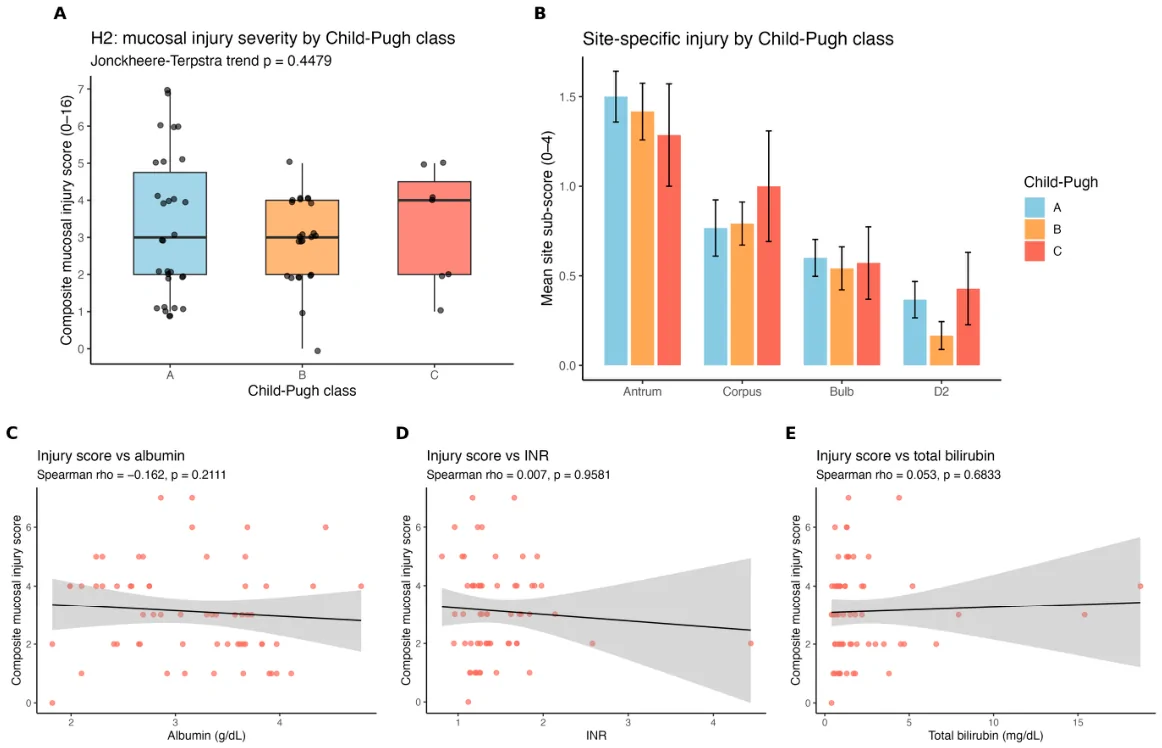
Relationship between mucosal injury and hepatic disease severity in cirrhotic patients (n = 61). (**A**) Composite mucosal injury score by Child–Pugh class (A, *n* = 30; B, *n* = 24; C, *n* = 7). Points represent individual patients; the horizontal line within each box denotes the median, box limits denote the interquartile range, and whiskers extend to the most extreme values within 1.5× the interquartile range; trend assessed by the Jonckheere–Terpstra test (*p* = 0.448). (**B**) Mean site-specific injury sub-scores by Child–Pugh class; error bars represent the standard error of the mean. (**C**–**E**) Scatterplots of composite injury score versus serum albumin (**C**), international normalized ratio (**D**), and total bilirubin (**E**), with linear fit and 95% confidence band; correlations assessed by Spearman’s rank correlation coefficient.

Spearman’s rank correlation coefficients between the composite injury score and serum albumin (ρ = −0.16, *p* = 0.21), INR (ρ = 0.01, *p* = 0.96), and total bilirubin (ρ = 0.05, *p* = 0.68) were all close to zero and non-significant, meaning that patients with worse synthetic function (lower albumin, higher INR, higher bilirubin) did not show correspondingly higher injury scores (Figure 4).

An internal validity check was performed: albumin, INR, total bilirubin, and hemoglobin were each compared across Child–Pugh classes to confirm that these laboratory markers behaved as clinically expected. All four showed significant, expected differences across classes (all *p* < 0.05), with albumin decreasing and INR and bilirubin increasing progressively from class A to class C (Table 5). This confirms that the Child–Pugh classification and laboratory data in this cohort were internally consistent and behaved as they should, and that the null finding described above is therefore specific to the mucosal injury score itself, rather than reflecting a data quality issue or a miscoded Child–Pugh variable.

### 3.5. Concurrent H. pylori Infection Shows a Positive Multiplicative Interaction with Cirrhosis on Mucosal Injury (Hypothesis 3)

To test the third hypothesis, patients were stratified into four mutually exclusive groups defined jointly by cirrhosis and *H. pylori* status: control/HP− (*n* = 73), control/HP+ (*n* = 28), cirrhosis/HP− (*n* = 49), and cirrhosis/HP+ (*n* = 12).

**Figure 5:**
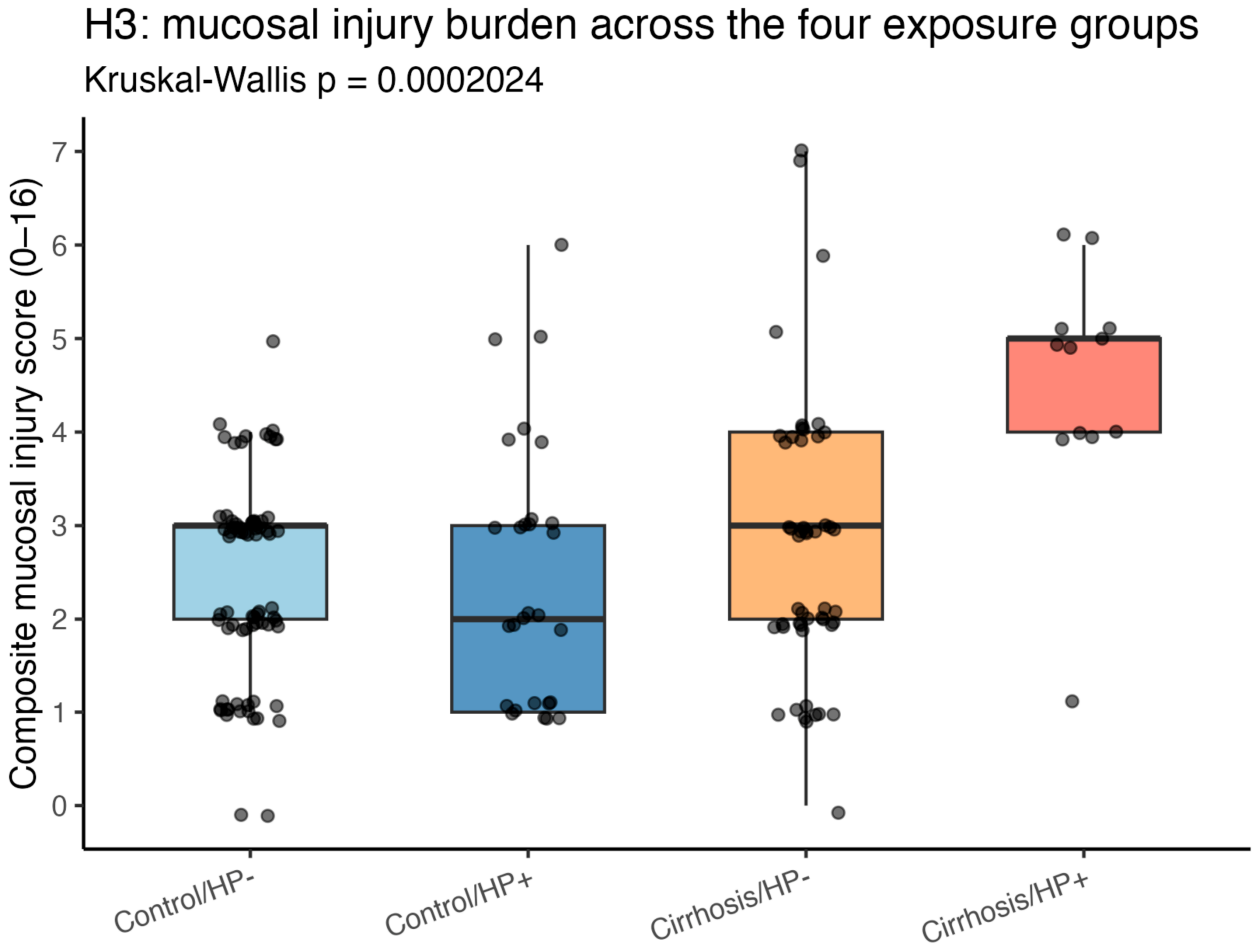
Composite mucosal injury score across cirrhosis × *H. pylori* exposure groups. Boxplots show the composite mucosal injury score in four groups defined jointly by cirrhosis and *H. pylori* status: Control/HP− (*n* = 73), Control/HP+ (*n* = 28), Cirrhosis/HP− (*n* = 49), Cirrhosis/HP+ (*n* = 12). Points represent individual patients; the horizontal line within each box denotes the median, box limits denote the interquartile range (25th–75th percentile), and vertical whiskers extend to the most extreme values within 1.5× the interquartile range from the box edges. Overall group comparison by the Kruskal–Wallis test (*p* = 0.0002); pairwise comparisons by Dunn’s test with Benjamini–Hochberg correction (Table 7).

Neither cirrhosis alone (cirrhosis/HP−) nor *H. pylori* alone (control/HP+) was associated with a meaningfully or statistically higher injury burden relative to unexposed controls (control/HP−); the mean injury scores in these three groups were closely similar, ranging narrowly from 2.41 to 2.78. It was only the co-exposed group, patients with both cirrhosis and concurrent *H. pylori* infection, which showed a markedly higher injury burden, with a mean score of 4.50, substantially above all three other groups (Table 6, Figure 5). This difference across the four groups was confirmed by the omnibus Kruskal–Wallis test (*p* = 0.0002), and Dunn’s post-hoc test, applied to identify which specific pairs of groups differed, showed that every pairwise comparison involving the co-exposed group reached significance (all adjusted *p* ≤ 0.0006), while none of the three remaining pairwise comparisons, among the control/HP−, control/HP+, and cirrhosis/HP− groups, reached significance (all adjusted *p* > 0.46). In other words, having cirrhosis alone, or *H. pylori* alone, did not distinguish a patient’s injury burden from that of an unexposed control, but having both conditions together did.

This descriptive four-group comparison was corroborated by a formal adjusted regression model built on the same four-group structure (reference category: control/HP−), which allows each group difference to be expressed as an incidence rate ratio with a confidence interval, rather than only as a significance test. In this model, only the cirrhosis/HP+ group showed a significantly increased injury burden relative to the reference group, with an adjusted of 1.87 (95% CI: 1.38–2.53, *p* < 0.0001), that is, an injury burden nearly double that of *H. pylori*-negative, non-cirrhotic controls. Neither the control/HP+ group (adjusted = 1.04, 95% CI: 0.79–1.37, *p* = 0.80) nor the cirrhosis/HP− group (adjusted = 1.15, 95% CI: 0.92–1.44, *p* = 0.22) differed significantly from the reference group (Table 7).

An exploratory formal interaction term (cirrhosis × *H. pylori*) was added to the regression model. This interaction term was itself statistically significant (=1.56, 95% CI: 1.03–2.38, *p* = 0.037).

## 4. Discussion

This study examined the burden and distribution of gastroduodenal mucosal injury in patients with liver cirrhosis compared with non-cirrhotic controls and tested three pre-specified hypotheses regarding the roles of cirrhosis, *H. pylori* infection, and hepatic disease severity. The discussion below addresses each hypothesis in turn, situating our findings within the existing literature on portal hypertensive gastropathy (PHG), *H. pylori*-associated gastritis, and cirrhosis-related gastrointestinal complications.

The baseline demographic and laboratory differences between groups were consistent with the expected clinical profile of cirrhosis. The higher proportion of male patients in the cirrhosis group mirrors the well-documented global sex disparity in cirrhosis burden, with men accounting for the majority of cirrhosis-related incident cases, deaths, and disability-adjusted life-years worldwide [15]. The lower hemoglobin levels in cirrhotic patients likely reflect anemia’s multifactorial pathogenesis in this population—chronic gastrointestinal blood loss (including from portal hypertensive gastropathy, the lesion most relevant here), hypersplenism, and iron, folate, or vitamin B12 deficiency [16]. Similarly, the higher INR and total bilirubin observed in cirrhotic patients are direct laboratory correlates of impaired hepatic synthetic and excretory capacity, consistent with reduced hepatic synthesis of coagulation factors and impaired bilirubin clearance described in cirrhosis [16]. These baseline differences confirm that the cirrhosis group in our cohort represented patients with clinically meaningful hepatic dysfunction, rather than early, biochemically silent disease, which is an important contextual consideration for interpreting the mucosal injury findings discussed below.

### 4.1. Cirrhosis Is Independently Associated with Corpus-Predominant Gastroduodenal Mucosal Injury (Hypothesis 1)

Our finding that cirrhotic patients carry a significantly higher gastroduodenal mucosal injury burden than non-cirrhotic controls, independent of *H. pylori* status and gastrotoxic medication use, is consistent with a substantial body of endoscopic literature describing cirrhosis as an independent risk factor for gastric mucosal damage [9,17]. In practical terms, this indicates that cirrhotic patients had, on average, an injury burden approximately 33% higher than controls, after accounting for *H. pylori* status and gastrotoxic medication use—that is, the excess injury seen in cirrhosis cannot be explained away by a higher prevalence of *H. pylori* infection or NSAID/antiplatelet/anticoagulant use in this group. Because the composite score aggregates lesion severity across four anatomically distinct sites, a global increase in this score could in principle reflect either a diffuse increase in injury at every site, or a more localized effect concentrated at one or a few sites. Importantly, the effect we observed was not diffuse across the upper gastrointestinal tract, but was concentrated almost exclusively in the gastric corpus, with no significant excess injury in the antrum, duodenal bulb, or second duodenal portion. This anatomical pattern strongly parallels the classic topography described for portal hypertensive gastropathy, which is well established in the literature to predominantly affect the gastric fundus and corpus rather than the antrum [18], and it reinforces the interpretation that the excess injury we observed in cirrhotic patients reflects, at least in part, a congestive/hemodynamic gastropathy-type process related to portal hypertension, superimposed on, but anatomically distinct from, the more antrum-predominant pattern typically associated with uncomplicated *H. pylori* gastritis.

This interpretation is further supported by comparison with our own reference dataset from the general endoscopic population, in which corporal erythema and corporal erosive gastritis were independent predictors of *H. pylori* infection (adjusted OR 1.75 and 2.24, respectively), whereas antral lesions—although more common overall—did not differ significantly between infected and uninfected patients [11]. Taken together, the two datasets suggest that, although the antrum remains the site most frequently and classically affected in *H. pylori*-associated gastritis, corporal mucosal changes may carry greater discriminative value for identifying both infection-related and cirrhosis-related injury specifically. This observation has direct practical relevance: endoscopists examining cirrhotic patients should not limit their attention to the antrum alone, but should systematically document corporal findings as well, given their stronger association with the mucosal injury patterns discussed here. 

Taken as a whole, Hypothesis 1 is statistically confirmed and anatomically well characterized: rather than a single global score, the site-specific breakdown identifies a candidate anatomical “signature” of cirrhosis-related injury. If confirmed in larger cohorts, a corpus-predominant, antrum-sparing pattern of erythema, erosions, or hemorrhage might raise clinical suspicion for underlying portal hypertension, warranting correlation with hepatic function tests and imaging. Given the single-center, cross-sectional design and modest sample size, this remains hypothesis-generating rather than an established diagnostic criterion.

### 4.2. Absence of a Correlation Between Mucosal Injury and Child–Pugh Class Diverges from Part of the Published Literature (Hypothesis 2)

Hypothesis 2 postulated that, among cirrhotic patients specifically, mucosal injury severity would increase progressively with worsening hepatic synthetic function, that is, that patients with more advanced cirrhosis (Child–Pugh class C) would show more severe endoscopic injury than those with better-preserved liver function (Child–Pugh class A). In contrast, our finding that the composite mucosal injury score did not increase progressively with Child–Pugh class, and did not correlate with albumin, INR, or total bilirubin, diverges from a substantial portion of the published literature on portal hypertensive gastropathy specifically. Several dedicated PHG cohorts have reported a significant association between PHG severity and Child–Pugh class: in a cross-sectional study of 404 patients with chronic liver disease, Tiwari et al. [18] found significant associations both between Child–Pugh class and the presence of PHG (*p* = 0.001) and between Child–Pugh class and PHG severity (*p* = 0.01), though notably that same study found no significant association between PHG and the Model for End-Stage Liver Disease (MELD) score. Similarly, in a large Korean cohort of 587 cirrhotic patients with directly measured hepatic venous pressure gradient (HVPG), Bang et al. [19] demonstrated that PHG grade correlated closely with HVPG, MELD score, serum albumin, and total bilirubin, that is, more severe hepatic dysfunction was associated with more severe gastropathy on a dose–response basis.

Several plausible explanations may account for this discrepancy. Most importantly, the cited studies used dedicated PHG severity grading systems (e.g., McCormack or Baveno), purpose-built to capture the congestive vasculopathic features (mosaic pattern, discrete red spots) that scale directly with portal pressure. Our composite score, by contrast, aggregated four lesion types (erythema/edema, erosions, ulcers, submucosal hemorrhage) across four sites into a single count-based outcome. Erosions and ulcers, in particular, are strongly influenced by local factors like *H. pylori* infection and gastrotoxic medication use (Hypothesis 1), which are not expected to track with Child–Pugh class. Mixing portal-hypertension-driven changes with *H. pylori* or medication-driven erosive changes may therefore have diluted a gradient that a dedicated PHG grade could have detected.

Second, the correlation with hepatic disease severity in the literature appears strongest when portal pressure is measured directly via HVPG, as in the study by Bang et al. [19], rather than estimated indirectly through the Child–Pugh score, which reflects hepatic synthetic function and clinical decompensation (ascites, encephalopathy) rather than portal pressure per se. As HVPG measurement was not available in our cohort, an acknowledged limitation of the present analysis, our study could only test the indirect relationship between synthetic liver function and mucosal injury, which may be inherently weaker and noisier than the direct hemodynamic relationship reported elsewhere.

Third, our Child–Pugh class C subgroup was small (*n* = 7 of 61), substantially limiting the power of both trend tests to detect a true gradient, particularly at the more advanced end of the spectrum where the effect would be expected to be most pronounced. A type II error therefore cannot be excluded, and this null result should be interpreted as inconclusive rather than definitive evidence against a Child–Pugh gradient.

Finally, our site-specific finding (Section 3.3) offers a biologically plausible account: portal pressure can already be substantially elevated at early, well-compensated cirrhosis (Child–Pugh A), well before synthetic function deteriorates to class B or C. If corpus injury develops once a pressure threshold is crossed, rather than scaling continuously with decompensation, this could explain the observed pattern—injury present across all classes but not progressively worse in advanced disease. This hypothesis, that mucosal injury tracks portal pressure rather than synthetic failure and may plateau early, is consistent with the pathophysiological model described by Zullo et al. [20] and more recently reviewed by Tan [21] and warrants testing with direct HVPG measurement.

In summary, while our null finding for Hypothesis 2 diverges from part of the dedicated PHG literature, this divergence is plausibly attributable to differences in outcome definition (composite injury score vs. dedicated PHG grade), the absence of direct portal pressure measurement, and limited statistical power in the most advanced disease subgroup, rather than indicating that no true relationship exists between portal hypertension and gastric mucosal injury in cirrhosis.

### 4.3. Concurrent H. pylori Infection Shows a Positive Multiplicative Interaction with Mucosal Injury in Cirrhotic Patients (Hypothesis 3)

The four-group analysis (Hypothesis 3) found that neither cirrhosis nor *H. pylori* alone significantly elevated injury burden relative to unexposed controls, whereas the co-exposed group—cirrhotic patients with concurrent *H. pylori* infection—showed markedly and significantly higher injury, with a formally significant interaction term. This pattern has direct implications for interpreting the existing, at times conflicting, literature on *H. pylori* and cirrhosis.

Literature on *H. pylori* and PHG in cirrhotic patients has been notably inconsistent: Sathar et al. [12] reported a significant association in 140 cirrhotic patients (OR = 2.13), whereas Noor et al. [13], studying 80 patients in Pakistan, found none (*p* = 0.080), concluding *H. pylori* does not cause or worsen PHG. Our four-group analysis offers a plausible explanation: if *H. pylori*’s effect on mucosal injury is conditional on underlying portal hypertension, with little independent effect in non-cirrhotic patients, studies modeling it purely as a main effect within cirrhotic-only samples—without testing for interaction or adequate co-exposed numbers—could easily reach inconsistent conclusions depending on cohort composition. Our significant interaction term (=1.56), despite a small co-exposed cell (*n* = 12), is to our knowledge among the few analyses to test this interaction formally, rather than only comparing *H. pylori*-positive and -negative subgroups.

This multiplicative-interaction finding is further supported by Abdel-Razik et al. [22], who found substantially higher pro-inflammatory and pro-angiogenic mediators (CRP, TNF-α, IL-6, VEGF) in H. pylori-positive versus -negative cirrhotic patients, alongside higher rates of PHG and gastric antral vascular ectasia. A plausible mechanism: portal hypertension already impairs gastric corpus microcirculation and defensive capacity (mucosal hypoxia, capillary congestion), onto which H. pylori’s inflammatory and ulcerogenic burden is superimposed, producing combined injury exceeding either insult alone—consistent with a model of combined vascular and inflammatory injury proposed for other gastric complications of portal hypertension [20].

*H. pylori*’s role in cirrhosis may not be uniformly detrimental across all clinical endpoints. In a prospective cohort of 159 cirrhotic patients with variceal bleeding, *H. pylori* infection was associated with a lower risk of rebleeding, attributed to atrophic changes reducing gastric acid output [23]. This suggests divergent effects depending on the outcome considered: harmful for baseline corpus mucosal inflammation and injury burden (our finding and that of Abdel-Razik et al. [21]), yet potentially protective for variceal hemorrhage, more directly related to acid secretion than mucosal inflammation. This is an important nuance: *H. pylori* eradication recommendations should not be generalized across mechanistically distinct endpoints; the specific clinical question should guide whether *H. pylori* is viewed as a risk factor or as incidentally protective.

This finding’s relevance is nonetheless tempered by its exploratory nature. The co-exposed subgroup was small (*n* = 12); while consistency across three independent approaches—the descriptive comparison, adjusted regression, and formal interaction term—strengthens confidence this is a genuine signal, replication in a larger, multicenter cohort is needed before this interaction can be considered established.

If confirmed, this would have an actionable clinical implication: *H. pylori* eradication may be of particular value in cirrhotic patients specifically, where its contribution to mucosal injury appears elevated [24], rather than in the general population, where our reference dataset and others show a more modest, additive effect [11].

### 4.4. Strengths and Limitations

This study has some limitations that should be considered when interpreting these findings. First, the interaction analysis for Hypothesis 3 relied on a co-exposed cell of only 12 patients, limiting the precision of the interaction estimate (95% CI: 1.03–2.38); this finding should therefore be regarded as exploratory and hypothesis-generating, warranting confirmation in a larger, ideally multicenter cohort. Second, hepatic venous pressure gradient (HVPG) was not measured in this cohort, restricting our ability to directly test the relationship between portal pressure and mucosal injury severity in Hypothesis 2. Third, the Child–Pugh class C subgroup was small (*n* = 7 of 61 cirrhotic patients), limiting statistical power to detect a true gradient across disease severity. Fourth, because the composite injury score was derived retrospectively from written endoscopy reports rather than recorded in real time using a structured proforma, the absence of an explicit mention of a given lesion type at a given site in the original report was coded as absence of that lesion; this may have led to underestimation of true injury burden if lesions were present but not explicitly documented in the original examination report. Fifth, the composite injury score was developed for this study and has not been externally validated; inter-rater reliability was not formally assessed, and equal weighting across lesion types was a simplifying assumption rather than an evidence-based severity scale. Sixth, the control group consisted of hospitalized patients undergoing endoscopy for clinical indications rather than asymptomatic or community-based individuals; the specific indications for endoscopy were not systematically coded in a structured format permitting formal statistical comparison between groups, given the retrospective, chart-based design. Seventh, given the predominance of alcohol-related etiology in our cirrhosis cohort, the generalizability of these findings to cirrhosis of other etiologies (viral, metabolic) remains to be confirmed in future studies with more balanced etiological representation. Eighth, markers directly reflecting portal hypertension severity, including the presence and grade of esophagogastric varices, formal portal hypertensive gastropathy grading, platelet count and splenomegaly on imaging, were not consistently documented across all medical records in a format suitable for quantitative analysis and could therefore not be incorporated into the present models.

Ninth, patients with known prior *H. pylori* eradication were excluded from this cohort; however, recent antibiotic or bismuth exposure not associated with formal eradication therapy was not systematically recorded for all patients. Proton pump inhibitor use, while recorded, can reduce bacterial density on standard staining; this risk was mitigated by the use of immunohistochemistry in cases of persistent inflammation without visible organisms, which can detect *H. pylori* antigen despite reduced bacterial density, though residual misclassification from unrecorded antibiotic exposure cannot be fully excluded. Tenth, separating gastrotoxic medication use into NSAIDs, antiplatelet agents, anticoagulants, and PPIs, and additionally adjusting for age, sex, smoking, alcohol use, and comorbidities, did not materially change the point estimate for the independent effect of cirrhosis, though this fully adjusted model showed borderline significance (*p* = 0.059), reflecting reduced power in a more heavily parameterized model. Eleventh, a sensitivity analysis restricted to cirrhotic patients without alcohol-related etiology (*n* = 9) no longer showed a significant cirrhosis effect (adjusted = 1.26, *p* = 0.20); given the small size of this subgroup, this finding should be interpreted as inconclusive due to limited statistical power rather than as evidence against an effect in non-alcoholic cirrhosis specifically.

Finally, the cross-sectional, single-center design limits causal inference and generalizability to other populations.

## 5. Conclusions

The pattern of results across all three hypotheses converges on a coherent overall narrative: cirrhosis is associated with a specific, corpus-predominant pattern of gastric mucosal injury that is compatible with portal hypertension-related mechanisms rather than global hepatic synthetic failure and this injury shows a significant positive multiplicative interaction with concurrent *H. pylori* infection, though this test cannot establish departure from additivity. These findings suggest that *H. pylori*’s association with mucosal injury in cirrhotic patients may be conditional on, and warrants further investigation regarding the presence of underlying portal hypertension.

## Figures and Tables

**Table 1:** Baseline demographic, clinical, and laboratory characteristics of cirrhotic patients and non-cirrhotic controls.

Characteristic	Controls (*n* = 101)	Cirrhosis (*n* = 61)	*p*
Age, years, mean (SD)	59.7 (15.5)	65.0 (9.7)	0.037
Male sex, *n* (%)	39/101 (38.6%)	42/61 (68.9%)	<0.001
Hemoglobin, g/dL, mean (SD)	12.2 (2.4)	10.4 (2.4)	<0.001
INR, mean (SD)	1.11 (0.32)	1.45 (0.51)	<0.001
Total bilirubin, mg/dL, mean (SD); median [IQR]	0.75 (0.45); med 0.60 [IQR 0.40–0.90]	2.22 (3.16); med 1.30 [IQR 0.80–2.20]	<0.001
*H. pylori* positive, *n* (%)	28/101 (27.7%)	12/61 (19.7%)	0.267
NSAID/antiplatelet/anticoagulant use, *n* (%)	44/101 (43.6%)	17/61 (27.9%)	0.065
Composite injury score, mean (SD); median [IQR]	2.44 (1.19); med 2 [IQR 1–3]	3.11 (1.61); med 3 [IQR 2–4]	0.0099

**Table 2:** Multivariate negative binomial regression model for composite gastroduodenal mucosal injury score.

Predictor	aCR	95% CI	*p*
Cirrhosis (vs. control)	1.33	1.09–1.63	0.006
*H. pylori* positive	1.25	1.01–1.54	0.037
NSAID use	1.09	0.78–1.53	0.62
Antiplatelet use	0.96	0.74–1.24	0.74
Anticoagulant use	0.97	0.74–1.28	0.84
PPI use	0.96	0.79–1.18	0.70

**Table 3:** Site-specific gastroduodenal mucosal injury sub-scores by cirrhosis status (adjusted IRR, Benjamini–Hochberg corrected).

Site	Control Mean	Cirrhosis Mean	Adjusted IRR	*p* (Corrected)
Antrum	1.22	1.44	1.22	0.34
Corpus	0.27	0.80	3.04	<0.0001
Bulb	0.66	0.57	0.86	0.64
D2	0.29	0.30	1.03	0.93

**Table 4:** Individual gastroduodenal lesion components by anatomical site and cirrhosis status.

Lesion	Control (*n* = 101)	Cirrhosis (*n* = 61)	OR (adj.)	95% CI	*p* (BH-Corrected)
Antral erythema	88/101 (87.1%)	53/61 (86.9%)	1.08	0.43–2.87	0.88
Antral erosions	26/101 (25.7%)	24/61 (39.3%)	2.06	1.03–4.16	0.19
Antral ulcers	5/101 (5.0%)	4/61 (6.6%)	1.37	0.35–5.16	0.75
Antral submucosal hemorrhage	4/101 (4.0%)	7/61 (11.5%)	3.05	0.91–11.49	0.25
Corpus erythema	15/101 (14.9%)	30/61 (49.2%)	5.96	2.81–13.32	<0.0001
Corpus erosions	4/101 (4.0%)	6/61 (9.8%)	2.49	0.71–9.58	0.40
Corpus ulcers	0/101 (0%)	0/61 (0%)	—	—	—
Corpus submucosal hemorrhage	8/101 (7.9%)	13/61 (21.3%)	2.91	1.16–7.67	0.16
Bulbar erythema	55/101 (54.5%)	29/61 (47.5%)	0.76	0.40–1.44	0.61
Bulbar erosions	4/101 (4.0%)	4/61 (6.6%)	1.67	0.41–6.85	0.61
Bulbar ulcers	6/101 (5.9%)	2/61 (3.3%)	0.59	0.10–2.45	0.61
Bulbar submucosal hemorrhage	2/101 (2.0%)	0/61 (0.0%)	0.33	0.002–4.57	0.61
D2 erythema	26/101 (25.7%)	17/61 (27.9%)	1.12	0.54–2.29	0.81
D2 erosions	3/101 (3.0%)	0/61 (0.0%)	0.19	0.001–2.10	0.40
D2 ulcers	0/101 (0%)	0/61 (0%)	—	—	—
D2 submucosal hemorrhage	0/101 (0.0%)	1/61 (1.6%)	6.92	0.35–1023.62	0.40

**Table 5:** Composite mucosal injury score and laboratory parameters by Child–Pugh class.

Variable	A (*n* = 30)	B (*n* = 24)	C (*n* = 7)	*p* (Kruskal–Wallis)
Injury score, mean (SD); median	3.23 (1.94); med 3	2.92 (1.14); med 3	3.29 (1.60); med 4	0.885
Albumin, g/dL, mean (SD)	3.34 (0.62)	3.03 (0.78)	2.65 (0.48)	0.017
INR, mean (SD)	1.41 (0.67)	1.45 (0.31)	1.62 (0.28)	0.021
Total bilirubin, mg/dL, mean (SD); median	1.18 (0.77); med 0.99	2.54 (3.31); med 1.40	5.60 (5.99); med 3.50	0.001
Hemoglobin, g/dL, mean (SD)	11.27 (2.16)	9.40 (2.19)	10.47 (2.53)	0.012

**Table 6:** Composite mucosal injury score across the four cirrhosis × *H. pylori* exposure groups.

Group	*n*	Mean Injury Score	Median
Control/HP−	73	2.41	3
Control/HP+	28	2.50	2
Cirrhosis/HP−	49	2.78	3
Cirrhosis/HP+	12	4.50	5

**Table 7:** Adjusted four-group model for composite mucosal injury score (reference: Control/HP−).

Comparison	Adjusted IRR	95% CI	*p*
Control/HP+ vs. Control/HP−	1.04	0.79–1.37	0.80
Cirrhosis/HP− vs. Control/HP−	1.15	0.92–1.44	0.22
Cirrhosis/HP+ vs. Control/HP−	1.87	1.38–2.53	<0.0001

## Data Availability

The original contributions presented in this study are included in the article. Further inquiries can be directed to the corresponding author.
